# Design and Performance of a Spatial 6-RRRR Compliant Parallel Nanopositioning Stage

**DOI:** 10.3390/mi13111889

**Published:** 2022-11-01

**Authors:** Ruizhou Wang, Heng Wu

**Affiliations:** 1 State Key Laboratory of Precision Electronic Manufacturing Technology and Equipment, Guangdong University of Technology, Guangzhou 510006, China; 2 Guangdong Provincial Key Laboratory of Cyber-Physical System, Guangdong University of Technology, Guangzhou 510006, China

**Keywords:** piezoelectric actuators, compliant parallel mechanisms, nanopositioning stages

## Abstract

Piezoelectric actuators (PEAs) and compliant parallel mechanisms (CPMs) are advantageous for designing nanopositioning stages (NPSs) with multiple degrees of freedom (multi-DOFs). This paper proposes a new NPS that uses PEAs and CPMs with multiple spatial DOFs. First, the design of the mechanism is introduced. Six parallel kinematics revolute-revolute-revolute-revolute (RRRR) branched chains were used to create a 6-RRRR CPM for superior mechanical performance. Three in-plane and three out-of-plane chains were combined using a two-in-one structure to ensure fabrication feasibility. A two-in-one 6-RRRR CPM was employed to build the proposed NPS. Second, the mechanical performance was analyzed. High-efficiency finite-element modeling approaches were derived using the compliance-based matrix method (CMM) and a pseudo-rigid body model (PRBM). The model included both 6-RRRR CPM and NPS. The simulation results validated the static and dynamic performance, and the experimental results verified the kinematics. Based on the newly designed mechanism and verified mechanical performance, the proposed 6-RRRR NPS contributes to the development of spatial multi-DOF NPSs using PEAs and CPMs.

## 1. Introduction

Piezoelectric-actuated NPSs with multiple degrees of freedom (multi-DOFs) are designed to locate, move, measure, fabricate, and manipulate objects with nanoscale precision and accuracy. One typical example is the piezoelectric-actuated in-plane 3-DOF (x, y, θz) NPS [1,2,3,4,5,6,7,8,9,10,11,12,13,14]. The corresponding applications include deep ultraviolet lithography [1], micro/nano manipulation [3,5,11], precise measurements [15,16], and precision machining [13]. Another widely applied example is piezoelectric-actuated spatial 3-DOF (x, y, z) NPSs [17,18,19,20,21,22,23,24,25,26,27,28,29,30,31,32]. The use of NPSs has been promoted in spatial nanoscale measurements and manipulation based on scanning probe microscopy (SPM) [18,21], particularly atomic force microscopy (AFM) [17,24,25,33,34]. Based on these two types of 3-DOF NPSs, NPSs with spatial 4-DOF (x, y, z, θz) or more DOFs have been developed [35,36,37,38,39,40].

For multi-DOF NPSs, the number of branched chains is not less than the number of DOFs. Depending on the arrangement of the mechanical branched chains, kinematic configurations of NPSs include CPMs, compliant serial mechanisms (CSMs), and compliant serial-parallel hybrid mechanisms (CHMs). The parallel-kinematic configuration, combined with equilateral symmetry and spatial geometry, limits thermal drift in position and orientation. This configuration holds a greater advantage for NPSs in that a high output stiffness, high payload, and excellent precision can be obtained simultaneously. As an example of in-plane 3-DOF (x, y, θz) NPSs, the 3-revolute-revolute-revolute (3-RRR) CPM has been proven to demonstrate advantageous mechanical performance, such as high rigidity and accuracy [1,3,4,5,6,9,10,11,12,13]. Many challenges with respect to the use of CPM to design spatial multi-DOF NPSs still need to be overcome.

In-plane CPMs can be easily fabricated monolithically using traditional mature approaches such as wire electrical discharge machining (WEDM) and ultra-precision computer numerical control (CNC) technology. The low fabrication cost of in-plane CPMs guarantees excellent mechanical performance. However, it is difficult to machine spatial CPMs independently with the appropriate or expected performance. Performance-prioritized spatial CPMs typically possess complex or bulky structures. Monolithic manufacturability is challenging [22,41]. Fabrication-prioritized spatial CPMs always decrease or sacrifice performance, because the fabrication feasibility and mechanical performance are in conflict with each other. Additive manufacturing technology was attempted using non-metallic materials [29]. However, metallic materials such as the aluminum alloys 7075 or 6061 are incompatible with current commercial 3D-printing devices. The manufacturing cost and mechanical performance of 3D-printed CPMs using metallic materials remain unsolved. Therefore, the problem of mechanism design to balance the fabrication feasibility and mechanical performance is the first challenge that needs to be overcome when designing spatial multi-DOF NPSs using CPMs.

Compared to the finite element method (FEM), the compliance-based matrix method (CMM) requires fewer nodes and frames. The CMM can be treated as a new balance between accuracy and efficiency, sacrificing little modeling accuracy to save computational time. Owing to the small number of frames, CMM is more sensitive to the modeling accuracy of every frame. The single-axis notch flexure hinge (SaNFH) has a simple structure and precise motion. In terms of the orientation of the sensitive revolving axis, SaNFHs can be divided into in-plane and out-of-plane types. The currently available compliance calculation equations are mainly focused on three in-plane DOFs. The out-of-plane compliance equations of in-plane SaNFHs are inadequate. The procedure of transferring non-hinge structures of the CPM into flexible beams is essential, but it cannot always avoid lending under simplified or equivalent conditions. This assumption also decreases the calculation accuracy and worsens the disadvantages of CMM. Therefore, analysis of the mechanical performance to balance the calculation accuracy and computational time is the second challenge when designing a spatial multi-DOF NPS based on CPM.

To balance the fabrication feasibility and mechanical performance, a probable solution would be to separate a spatial CPM into two independently fabricated CPMs. A spatial two-in-one 6-RRRR scheme was proposed to provide a new solution for solving the first challenge. To balance the calculation accuracy and computational time, a simplified equivalent analysis approach of the mechanical performance using the CMM was proposed to solve the second challenge. The contributions of this work are the mechanism design and mechanical performance, verified in Section 4 and Section 5.

## 2. Mechanism Design

This paper designs a spatial multi-DOF NPS for the micro manipulation inside a commercial scanning electron microscope (SEM). The external dimensional constraint of the NPS is less than ϕ200 × 60 mm${}^{3}$, to be embedded into the SEM chamber. The first-order natural frequency of the end-effector is above 700 Hz, and the frequency along the vertical DOF (z) is no less than 900 Hz. The workspace is more than 120 μm (x) × 150 μm (y) × 80 μm (z) × 5000 μrad (θz). The trajectory tracking accuracy of the end-effector is better than 60 nm.

### 2.1. 6-RRRR CPM for the Mechanics Performance

**Figure 1:**
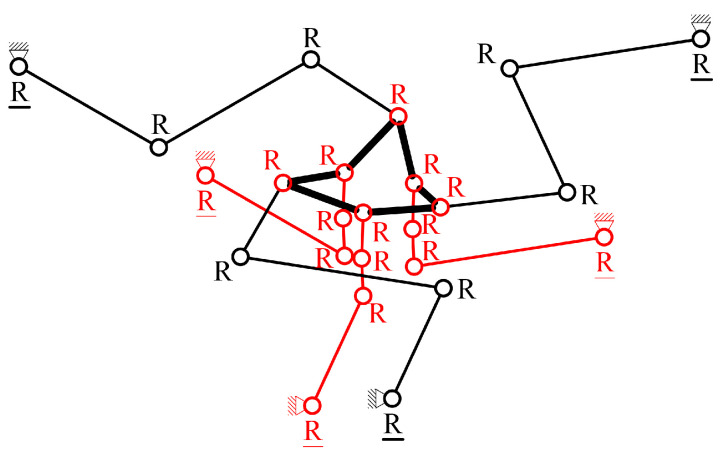
Configuration scheme of the new spatial 6-RRRR CPM.

A new CPM design scheme for a spatial multi-DOF NPS is proposed. The scheme contains six parallel-kinematic branched chains. Each branched chain is composed of four serially connected revolute joints. Six chains connect six different PEAs and one common end effector. The first revolute joint of each branched chain acts as the equivalent active joint, labeled as “R”. The other three revolute joints of the branched chain, marked using “RRR”, act as passive joints. The mechanism is then named 6-RRRR CPM. A brief schematic of the 6-RRRR CPM is shown in Figure 1.

In this paper, the term “plane” refers to the XOY plane in the Cartesian coordinate system by default. All plane-related concepts, such as “in-plane” and “out-of-plane”, refer to the default. As shown in Figure 1, the black circles represent in-plane revolute joints and the red circles represent out-of-plane joints. The black solid lines connecting two black circles represent in-plane flexible beams connecting two revolute joints. The black bold hexagon, composed of six bold solid lines and six red circles, represents the end effector. The red solid lines connecting two red circles represent out-of-plane flexible beams connecting two out-of-plane revolute joints.

The six chains of the 6-RRRR CPM are divided into three in-plane RRRR branched chains (marked in black) and three out-of-plane RRRR branched chains (marked in red). The end effector connects six branched chains independently using six different out-of-plane revolute joints.

#### 2.1.1. **R** Represents the **Revolute** Joint

**Figure 2:**
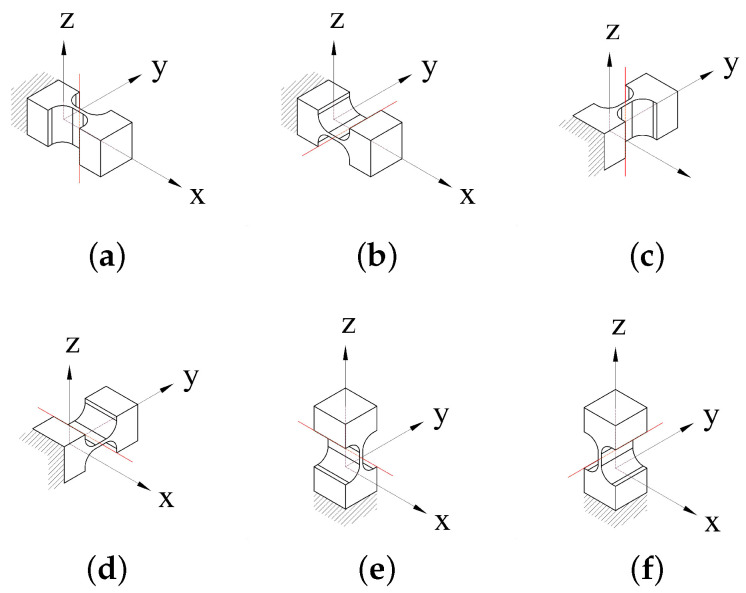
Definition of in-plane and out-of-plane revolute joints using SaNFHs. (**a**) In-plane **R**; (**b**) out-of-plane **R**; (**c**) in-plane **R**; (**d**) out-of-plane **R**; (**e**) out-of-plane **R**; (**f**) out-of-plane **R**.

An SaNFH has a simple mechanical structure and high rotary accuracy. To decrease the structural and modeling complexity, SaNFHs are employed as whole revolute joints of the 6-RRRR CPM. According to this definition, an SaNFH possesses only one unique sensitive revolving axis. The SaNFHs of the CPM contain three orientations (x, y, z) of the revolving axis and three directions (x, y, z) of axial elongation. In terms of revolving and elongation axes, SaNFHs are divided into six types. The aforementioned two types are known as in-plane SaNFHs. The other four types are out-of-plane SaNFHs. A description of the six SaNFHs is presented in Figure 2.

The in-plane compliance calculation equations for in-plane SaNFHs are sufficient and have the required high accuracy. In contrast, precise reference expression equations are either not available for out-of-plane modeling of in-plane SaNFHs or have poor calculation accuracy. The same problem exists for in-plane compliance calculation for out-of-plane SaNFHs.

#### 2.1.2. **R** Represents the Equivalent Active **Revolute** Joint

PEAs demonstrate high output force, nanometer-scale accuracy, and fast response. Typical PEAs produce only translational displacement rather than angular displacement. For the 6-RRRR CPM, the entire joint was a revolute joint. Therefore, an equivalent driving system is necessary. The translational displacement of the PEA was transferred to the rotational angle of the first revolute joint. However, a PEA subject to the sintering and bonding of multi-layer piezoelectric ceramic plates should not experience shear or lateral forces. Therefore, a driving system was designed to achieve the so-called “equivalent active **revolute** joint **R**”.

**Figure 3:**
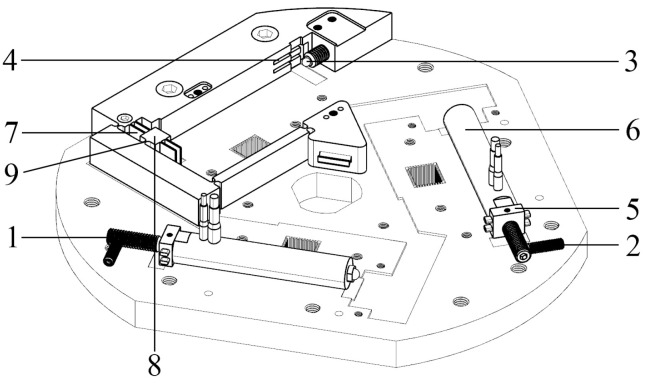
Components of the equivalent active **revolute** joint **R**. 1: First-level preload screw, 2: second-level preload screw, 3: steel ball, 4: guiding groove of a preload block, 5: preload block, 6: PEA, 7: SaNFH for displacement guiding, 8: input port, and 9: SaNFH for input displacement.

The first function of the driving system is to translate the nanometer-scale translational displacement into a small rotational angle. The second function is to maintain a continuous, stable, and safe connection during any nanometer-scale displacement-transfer procedure. The third function is to protect the PEA from shear/tensile stress, torsion/pitch, or lateral force/moment. The design scheme of the driving system is illustrated in Figure 3.

The driving system, which acts as an equivalent active **revolute** joint **R**, is shown in Figure 3. The driving system is composed of a PEA, a preload-guiding subsystem, and an input-guiding subsystem. The preload-guiding subsystem contains the first-level preload screw, second-level preload screw, steel ball, preload block, and guiding groove of the preload block. The input-guiding subsystem consists of a guiding flexure hinge, input port, and flexure hinge for the displacement input.

PEAs generate nanometer-resolution displacement. Both the preload-guiding subsystem and input-guiding subsystem guarantee nanometer-scale movement along a straight line. The flexible beam that connects the input flexure hinge is moved by the transferred movement. The first SaNFH produces a small rotational angle. The entire procedure can be treated as equivalent movement produced by the first SaNFH. Subsequently, the SaNFH is labeled as **R**. The other three serial-connected SaNFHs of the same branched chain are denoted using **RRR**, referred to as three passive revolute joints.

### 2.2. Two-in-One Structure for Fabrication Feasibility

Three requirements need to be fulfilled to ensure fabrication feasibility. First, it should be possible to fabricate the prototype using currently available machines. Second, the fabrication cost should be reasonable such that fabrication is within reach of most users. Third, despite the reduction in the fabrication cost, mechanical performance should be guaranteed.

A two-in-one structure is proposed for feasibility of fabrication. The structure consists of upper and lower sub-structures. The upper substructure has three in-plane branched chains, which are seen as an upper or in-plane 3-RRRR CPM. The lower substructure has three out-of-plane chains, which are considered to be a lower or out-of-plane 3-RRRR CPM.

**Figure 4:**
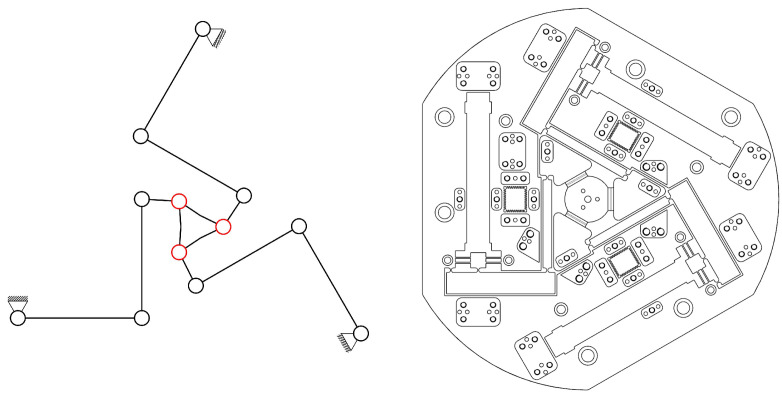
Upper sub-structure of the two-in-one structure.

The upper substructure of the two-in-one structure was an in-plane 3-RRRR CPM. Similar to traditional 3-RRR CPMs [1,3,4,5,6,9,10,11,12,13], the proposed 3-RRRR CPM has low manufacturing cost and high mechanical performance. This structure therefore satisfies the three requirements for fabrication feasibility. The upper substructure could be fabricated monolithically, as shown in Figure 4.

**Figure 5:**
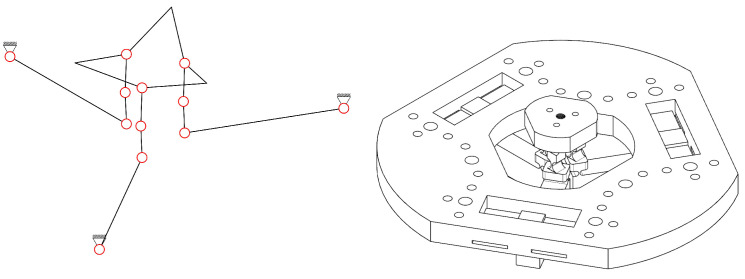
Lower sub-structure of the two-in-one structure.

The lower substructure of the two-in-one structure is an out-of-plane 3-RRRR CPM. The substructure can be fabricated monolithically, as shown in Figure 5.

The two substructures are connected using a metal plate and several fasteners to form a sandwich structure with the two substructures constituting two slices of bread and the metal plate the filling. The upper substructure is characterized by both the out-of-plane compliance of the in-plane SaNFHs and in-plane compliance of the out-of-plane SaNFHs. The lower substructure is only described by the in-plane compliance equations of the out-of-plane SaNFHs.

### 2.3. 6-RRRR NPS Using the Two-in-One 6-RRRR CPM

#### 2.3.1. Actuators and Sensors for Transforming the CPM into an NPS

Based on the 6-RRRR CPM, six PEAs were selected to drive six parallel kinematic RRRR branched chains. Three packaged PEAs are located within the upper in-plane 3-RRRR CPM. The other three naked PEAs are embedded in the lower out-of-plane 3-RRRR CPM. The PEAs are arranged symmetrically axially and located at a fixed base. The setup reduces the active moving mass and increases the loading capacity. Four capacitive sensors are used to measure the displacements along the four DOFs of the end-effector.

**Figure 6:**
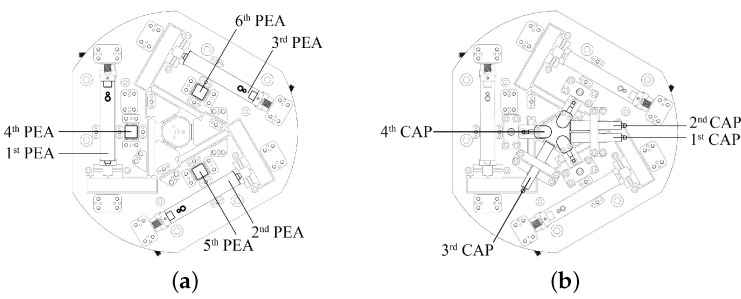
A 6-RRRR CPM, actuators and sensors to build a 6-RRRR NPS. (**a**) Six piezoelectric actuators; (**b**) four capacitive sensors.

The 6-RRRR CPM is defined as simply a mechanism without actuators or sensors. The 6-RRRR NPS was built by equipping the CPM with actuators and sensors, as shown in Figure 6. The motion controller of the NPS includes two levels of servo control loop. The first loop originates from the driving system. Three strain gauge sensors (SGSs), embedded into the internal space of three packaged PEAs, measure the elongation of the PEAs. The measured results of the SGSs act as the displacement feedback of the first loop. The second loop is situated at the end effector. Four capacitive sensors provide the measured results of the end-effector to build the second loop.

#### 2.3.2. Overall Design

Another connecting plate was employed to examine the performance of the 6-RRRR NPS. The plate is used to attach the NPS to a vibration-isolation table and provides installation space for the lower substructure.

**Figure 7:**
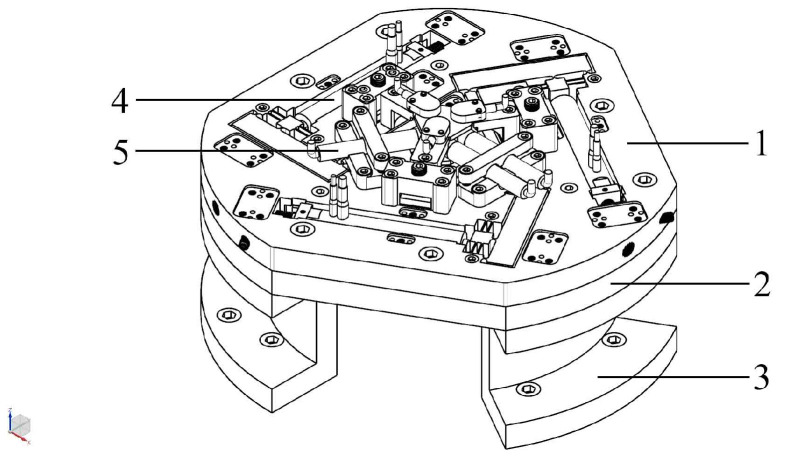
Proposed 6-RRRR NPS mainly using the 6-RRRR CPM. 1: Upper sub-structure; 2: connecting plate between the two sub-structures; 3: connecting plate between the NPS and the vibration-isolation table; 4: actuators; 5: sensors.

The lower substructure is not shown in Figure 7, as it is shielded by the upper substructure. The upper connecting plate shown in Figure 7 and labeled as “2’’, serves to hold together the two-in-one structure. The lower connecting plate, labeled “3’’, provides an installation space for the lower substructure.

## 3. Mechanics Performance

The statics, dynamics, and kinematics were analyzed to evaluate the mechanical performance. First, the input stiffness of the six parallel-kinematic branched chains, input coupling ratio among the six chains, output stiffness of the end-effector, and output coupling ratio along four DOFs were selected as four indexes of the static performance. Second, the natural frequency was employed to represent the dynamic performance. Third, the workspace and trajectory tracking precision were used as two indices of the kinematic performance.

Six PEAs were added to the 6-RRRR CPM to construct the 6-RRRR NPS. The CPM does not contain any PEAs, only the mechanism of the six RRRR chains and one end effector. However, for the NPS, the equivalent stiffnesses of the PEAs must be considered. The three in-plane PEAs were processed as elastic supports with a stiffness of 19 N/μm and the three out-of-plane PEAs with a stiffness of 98 N/μm. The CPM and NPS exhibited different mechanical performances.

The theoretical derivation of the static performance focuses on the 6-RRRR CPM rather than on the 6-RRRR NPS. The results reflect the relationship between the forces and displacements without taking into account the PEAs. The dynamics are focused on both the CPM and NPS. A comparison is presented to show the impact of PEAs. The kinematics, which focus on the NPS rather than the CPM, reflect the evaluation of the PEA-actuated movement. The PEAs play an important role in movement, and because the CPM does not contain any PEAs, it is therefore neglected.

### 3.1. Static Performance

The static modeling approach is an analysis and provides an evaluation foundation prior to the analysis of the dynamics and kinematics. The equivalent stiffness of the statics is a key parameter for deriving the stiffness matrix of dynamics. The stiffness and coupling ratio of the statics are essential elements of kinematics. A CMM was applied to calculate the static performance.

#### 3.1.1. Statics Model Using the CMM

Similar to the previous two-in-one mechanical structure, the analysis model was also divided into two steps for the two substructures. First, the three in-plane parallel-kinematic branched chains of the 6-RRRR CPM were analyzed.

**Figure 8:**
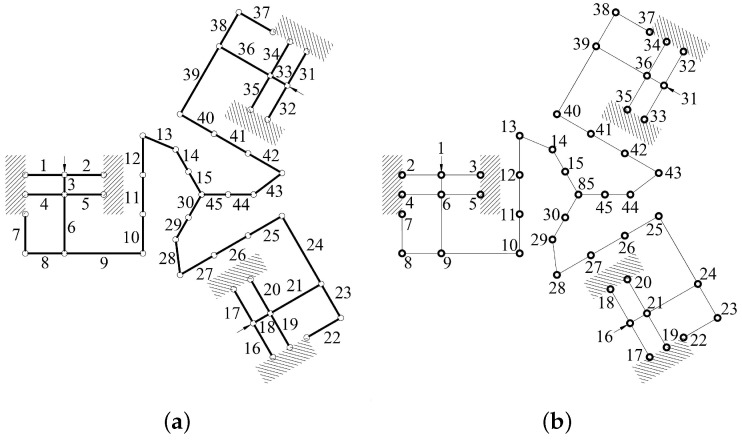
Simplified equivalent statics model of three in-plane branched chains. (**a**) Frames; (**b**) nodes.

Twelve right-circular SaNFHs, three corner-filled SaNFHs, and 30 flexible beams were used. Forty-six nodes and forty-five frames were selected to build the 6-RRRR CPM. The divisiory nodes of the in-plane stiffness model are shown in Figure 8b. The nodes are denoted from 1 to 45 and 85. The three driving ports are represented by nodes 1, 16, and 31. The flexible frames of the stiffness model are presented in Figure 8a. The SaNFHs and beams are labeled from 1 to 45.

**Figure 9:**
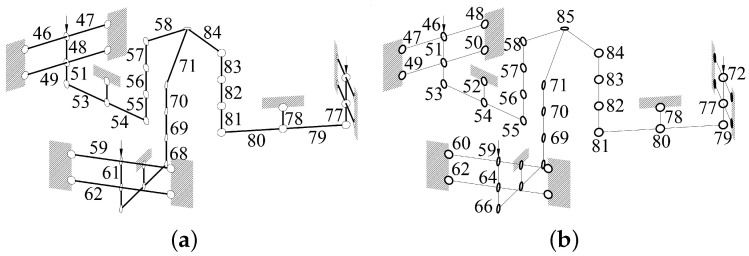
Simplified equivalent statics model of the three out-of-plane chains. (**a**) Frames; (**b**) nodes.

Second, the three out-of-plane parallel-kinematic branched chains were analyzed. This analysis involved nine right-circular SaNFHs, six corner-filled SaNFHs, and twenty-four flexible beams. The nodes and frames of the out-of-plane stiffness model are shown in Figure 9.

#### 3.1.2. Generic Formulation

**Figure 10:**
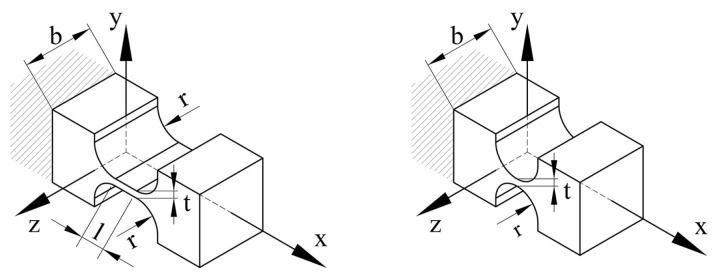
Corner-filleted and right-circular SaNFHs.

The frames were divided into two types: SaNFHs and beams, as shown in Figure 10. The calculation accuracy of the static performance mainly depends on the compliance of the SaNFHs.

**Figure 11:**
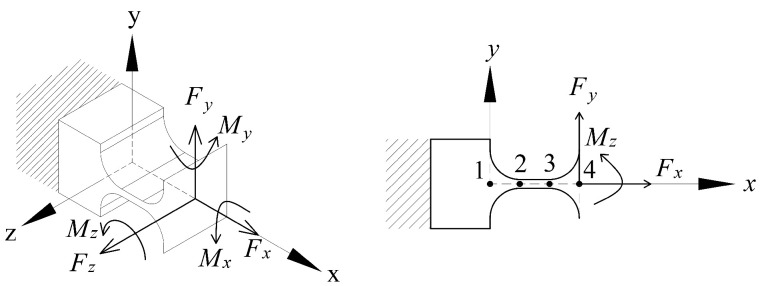
Deformation analysis of the corner-filleted SaNFH.

An SaNFH has a simple mathematical formula for the notch curve. Notches include right-circular, corner-filleted, straight-beam, filleted V-shaped, elliptic, parabolic, hyperbolic, and other user-defined curves. The right-circular SaNFH had accurate rotational motion. Corner-filled SaNFHs are characterized by high compliance and rotational accuracy, but with low stress levels. The right-circular SaNFH can be treated as a simplified corner-filled SaNFH (*l* = 0). The force and moment analysis is shown in Figure 11.

Owing to the three out-of-plane branched chains, the entire six-DOF closed-form compliance matrix is presented to describe the SaNFH of the 6-RRRR NPS. Three assumptions were made: linear stress-strain relationships for normal and tangential stresses, small elastic deformation, and an Euler-Bernoulli beam. The generic closed-form compliance matrix of the SaNFHs can then be described as follows:

(1) $$\left\{\begin{array}{c} \delta_{z}\\ \delta_{x}\\ \delta_{y}\\ \theta_{z} \end{array}\right\}=\left[\begin{array}{cccc} C_{\delta z-Fz} & 0 & 0 & 0\\ 0 & C_{\delta x-Fx} & 0 & 0\\ 0 & 0 & C_{\delta y-Fy} & C_{\delta y-Mz}\\ 0 & 0 & C_{\theta z-Fy} & C_{\theta z-Mz} \end{array}\right]\left\{\begin{array}{c} F_{z}\\ F_{x}\\ F_{y}\\ M_{z} \end{array}\right\}$$

where $\delta_{z}$, $\delta_{x}$, $\delta_{y}$, and $\theta_{z}$ represent the displacements of the deformation in the four DOFs; $F_{z}$, $F_{x}$, $F_{y}$, and $M_{z}$ represent the external forces and torques, and *C* represents the compliance matrix element.

(2) $$\begin{array}{cc} \; & C_{\delta z-Fz}=\int_{0}^{l+2r}x^{2}{\left[EI_{y}\left(x\right)\right]}^{-1}dx\\ \; & C_{\delta x-Fx}=\int_{0}^{l+2r}{\left[EA\left(x\right)\right]}^{-1}dx\\ \; & C_{\delta y-Fy}=\int_{0}^{l+2r}x^{2}{\left[EI_{z}\left(x\right)\right]}^{-1}dx\\ \; & C_{\delta y-Mz}=\int_{0}^{l+2r}x{\left[EI_{z}\left(x\right)\right]}^{-1}dx\\ \; & C_{\theta z-Fy}=\int_{0}^{l+2r}x{\left[EI_{z}\left(x\right)\right]}^{-1}dx=C_{\delta y-Mz}\\ \; & C_{\theta z-Mz}=\int_{0}^{l+2r}{\left[EI_{z}\left(x\right)\right]}^{-1}dx \end{array}$$

Based on the stiffness model presented in Figure 8 and Figure 9, the equation for the calculation of the stiffness is expressed as

(3) Kx=F

where K is the equivalent stiffness matrix and F is the external force matrix. The corresponding displacement deformation vector x is defined as follows:

(4) $$x={\left[\begin{array}{ccccc} q^{1T}, & \ldots , & q^{iT}, & \ldots , & q^{nT} \end{array}\right]}^{T}$$

where *i* represents the serial number of the node and $q^{i}$ is the displacement vector of the $i^{th}$ node, expressed as

(5) $$q^{i}={\left[\begin{array}{cccc} \delta z_{i} & \delta x_{i} & \delta y_{i} & \theta z_{i} \end{array}\right]}^{T},\;i=1,2,\ldots ,85$$

$j_{1}$ ($j_{1}\in \Omega_{1}$) represents the serial number of the ground node. $\delta z_{j_{1}}$, $\delta x_{j_{1}}$, $\delta y_{j_{1}}$, and $\theta z_{j_{1}}$ in Equation (5) corresponding to these nodes are set to zero. $\Omega_{1}$ is defined as follows:

(6) $$\begin{array}{c} \Omega_{1}=\left\{2,3,4,5,7,17,18,19,20,22,32,33,34,35,37\right.\\ \left.47,48,49,50,52,60,61,62,63,65,73,74,75,76,78\right\} \end{array}$$

#### 3.1.3. Calculation of Stiffness

$j_{2}$ is defined to represent the serial number of nodes at the six input ports of the six parallel-kinematic branched chains. $k_{in}$ represents the input stiffness of the 6-RRRR CPM. The input stiffness at node $j_{2}$ was obtained using

(7) $$k_{in}^{j_{2}}=F_{j_{2}}/\delta_{j_{2}},j_{2}\in \Omega_{2},\Omega_{2}=\left\{1,16,31,46,59,72\right\}$$

where $F_{j_{2}}$ indicates that a force of 100 N is applied to node $j_{2}$, and $\delta_{j_{2}}$ represents the corresponding total translational displacement at node $j_{2}$.

The output stiffness along the three translational DOFs of the end-effector reflects the characteristics of the 6-RRRR CPM and NPS. $j_{3}$ represents the DOF of the end-effector. $k_{out}$ represents the output stiffness, which along DOF $j_{3}$, was solved using

(8) $$k_{out}^{j_{3}}=F_{j_{3}}/\delta_{j_{3}},j_{3}\in \Omega_{3},\Omega_{3}=\left\{z,x,y,\theta z,\theta x,\theta y\right\}$$

where $F_{j_{3}}$ indicates that a force of 100 N is applied to the end effector along the translational DOF $j_{3}$ or a moment of 3000 N·mm along the rotational DOF $j_{3}$.

#### 3.1.4. Calculation of Coupling Ratios

Modeling the mechanical performance and controller design of the 6-RRRR NPS becomes complex because of the coupling of the 6-RRRR CPM. The coupling occurs at both the six input ports and six output DOFs of the end effector. The coupling characteristics were expressed using the input and output coupling ratios, respectively.

$j_{21}$ and $j_{22}$ represent two different nodes of $j_{2}$. $c_{in}$ represents the input-coupling ratio. Only one parallel kinematic chain is actuated. The other five chains had no active input displacements; instead, they had passive displacements. The input coupling ratio is defined as follows:

(9) $$c_{in}^{j_{21}j_{22}}=\delta_{j_{22}}/\delta_{j_{21}}\times 100\%,j_{21},j_{22}\in \Omega_{2},j_{21}\neq j_{22}$$

where $\delta_{j_{21}}$ represents the active displacement along the input port $j_{21}$, $\delta_{j_{22}}$ represents the passive displacement along port $j_{22}$, and $c_{in}^{j_{21}j_{22}}$ indicates that coupling deformation occurred in port $j_{22}$ because of the actuation in port $j_{21}$.

$j_{31}$ and $j_{32}$ represent two different DOFs of $j_{3}$. $c_{out}$ represents the output-coupling ratio. Only one DOF of the end effector was actuated. The other five DOFs exhibited no active displacements and underwent passive displacements instead. The output coupling ratio is defined as follows:

(10) $$c_{out}^{j_{31}j_{32}}=\left|\delta_{j_{32}}/\delta_{j_{31}}\right|\times 100\%,j_{31},j_{32}\in \Omega_{3},j_{31}\neq j_{32}$$

where $\delta_{j_{31}}$ denotes the active output displacement along the DOF of $j_{31}$, $\delta_{j_{32}}$ represents the passive displacement along the DOF of $j_{32}$, and $c_{out}^{j_{31}j_{32}}$ indicates that coupling deformation occurred along $j_{32}$ because of actuation along $j_{31}$.

### 3.2. Dynamics Performance

The dynamic model was developed based on two simplified conditions: The first condition was that only four prior DOFs (z, x, y, θz) were considered. In the second condition, the damping elements are neglected. The model was simplified into a pseudo-rigid body model (PRBM) with twenty-four elastic springs and 18 rigid beams. The equivalent mass and stiffness matrices are specified relative to the end effector.

**Figure 12:**
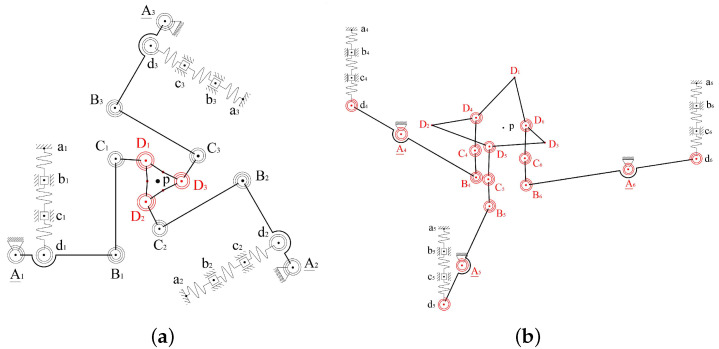
Equivalent dynamics model based on the two simplified conditions. (**a**) In-plane 3-RRRR; (**b**) out-of-plane 3-RRRR.

As shown in Figure 12, the black springs represent the in-plane revolute joints, and the red springs represent the out-of-plane joints. The natural frequencies were derived using:

(11) $$M{\left[{\ddot{z}}_{p}\;{\ddot{x}}_{p}\;{\ddot{y}}_{p}\;{\ddot{\theta }}_{zp}\right]}^{T}+K{\left[z_{p}\;x_{p}\;y_{p}\;\theta_{zp}\right]}^{T}=0$$

### 3.3. Kinematics Performance

**Figure 13:**
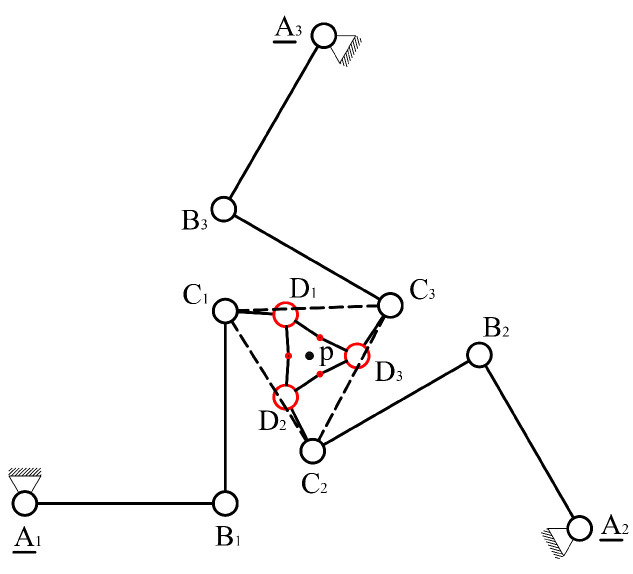
Equivalent kinematics model based on two simplified conditions.

A kinematic model was developed based on two simplified conditions, as shown in Figure 13. The first condition is that the three-fourth-revolute joints of the three in-plane chains are neglected. The second condition is that both the third and fourth revolute joints in every out-of-plane chain are neglected.

(12) $$J_{p}{\left[{\dot{z}}_{p}\;{\dot{x}}_{p}\;{\dot{y}}_{p}\;{\dot{\theta }}_{zp}\right]}^{T}=J_{\delta_{PEA}}{\left[{\dot{\delta }}_{j21}\;{\dot{\delta }}_{j22}\;{\dot{\delta }}_{j23}\;{\dot{\delta }}_{j24}\right]}^{T}$$

The forward-kinematic Jacobian matrix *J* is derived to calculate the workspace. An inverse-kinematic Jacobian matrix *J*′ was derived for trajectory tracking. *J* and *J*′ are given by

(13) $$J=J_{\delta_{PEA}}/J_{p},\;J^{\prime }=J_{p}/J_{\delta_{PEA}}$$

Each point in the workspace has a different *J* value. Although every point along the trajectory also has a different *J*′, a constant matrix of *J*′ is employed to decrease the computational time of a single servo cycle. Based on these two simplified conditions, the derivation is the same as that of the traditional 3-RRR NPSs [3,5,9,10]. The detailed procedure is not presented in this paper.

## 4. Verification of the Statics and Dynamics Performance

Mathematical equations for the static performance were derived using the CMM. The dynamic performance was calculated based on the PRBM. FEM was employed to examine the calculation accuracy of the CMM and PRBM. ANSYS, a powerful commercial software base on the FEM, was employed to verify the static and dynamic performance.

In addition to the verification, other indices were also provided. The results describe the static and dynamic performance in more detail. The indices were used for comparison with other spatial multi-DOF NPSs.

### 4.1. Verification of the Statics Performance

Three indices were presented to examine the proposed approaches to analyze the static performance. The percentage errors are provided to show the calculation accuracy. The theoretical analysis of the statics focuses on the 6-RRRR CPM. The verification focused on the CPM to demonstrate the calculation accuracy. The performance indexes of the 6-RRRR NPS are also provided in more detail.

#### 4.1.1. Input Stiffness

First, the input stiffness along each parallel kinematic chain was examined. The results were calculated using CMM and FEM. A comparison is presented in Table 1.

#### 4.1.2. Output Stiffness

Second, the output stiffness of the 6-RRRR CPM is provided. Two boundary conditions were considered in this study. When the six input ports are free, the output stiffness exhibits minimum values. If the six input ports are set as fixed supports, then the output stiffness reaches its maximum value. Both results can be calculated using the FEM and CMM. A comparison is presented in Table 2 and Table 3.

As presented in Table 2, the minimum output stiffness means that the six input ports are free, or PEAs are not considered. The maximum output stiffness means that the six input ports are restrained to be stationary, or the six PEAs possess infinitely large stiffnesses. The maximum values cannot be obtained, but show a clear boundary.

#### 4.1.3. Input Coupling Ratio

Third, the input coupling ratios of 6-RRRR CPM and NPS were examined. The results are presented in Table 4.

As presented in Table 5, three in-plane axes using 35.0 μm, and out-of-plane using 40.0 μm.

#### 4.1.4. Output Coupling Ratio

Finally, the output coupling ratios are inspected, as shown in Table 6 and Table 7. Relative to the input coupling ratio, the output coupling ratio focuses on the end-effector of the 6-RRRR CPM and NPS.

#### 4.1.5. Maximum Stress

The aluminum alloy, AA 7075-T651, possessing a standard yield strength of 503 MPa (73.0 ksi) and a safety factor of 1.50–2.20, was selected as the material for the 6-RRRR CPM. The maximum nominal elongations of the in-plane PEAs were set to 45.0 μm, and the maximum elongations of the out-of-plane PEAs were 42.9 μm.

The maximum stress over the entire workspace was 163 MPa. Therefore, the safety factor was 3.10, satisfying the allowable standard AA 7075-T651.

### 4.2. Verification of the Dynamics Performance

**Figure 14:**
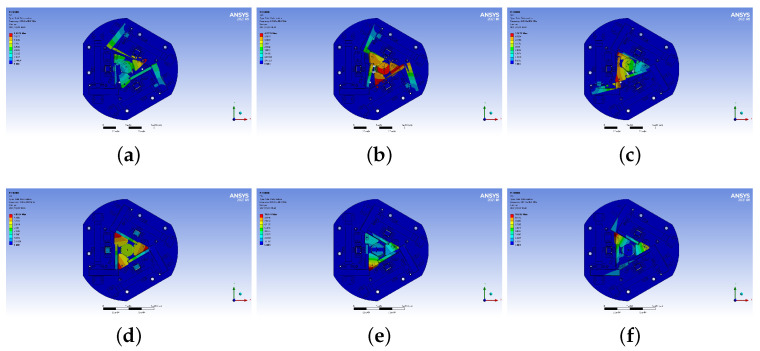
Natural frequencies of the 6-RRRR CPM. (**a**) 1st, $4.00\times 10^{2}$ Hz; (**b**) 2nd, $4.26\times 10^{2}$ Hz; (**c**) 3rd, $4.60\times 10^{2}$ Hz; (**d**) 4th, $4.78\times 10^{2}$ Hz; (**e**) 5th, $8.95\times 10^{2}$ Hz; (**f**) 6th, $1.05\times 10^{3}$ Hz.

The modal parameters of the 6-RRRR CPM without actuation and load are shown in Figure 14.

The first three modes occur in the three in-plane DOFs and the fourth mode in the out-of-plane translational DOF. By specifying the six input ports of the 6-RRRR CPM as free ends and by setting the end-effector to no load, the natural frequencies are minimized. By setting the six input ports as fixed ends, the frequencies are maximized. For the 6-RRRR NPS, the input ports were set as the equivalent stiffnesses of the PEAs. The results are shown in Table 8.

## 5. Experimental Validation of the Kinematics Performance

### 5.1. Experimental Setup

#### 5.1.1. Prototype Fabrication

**Figure 15:**
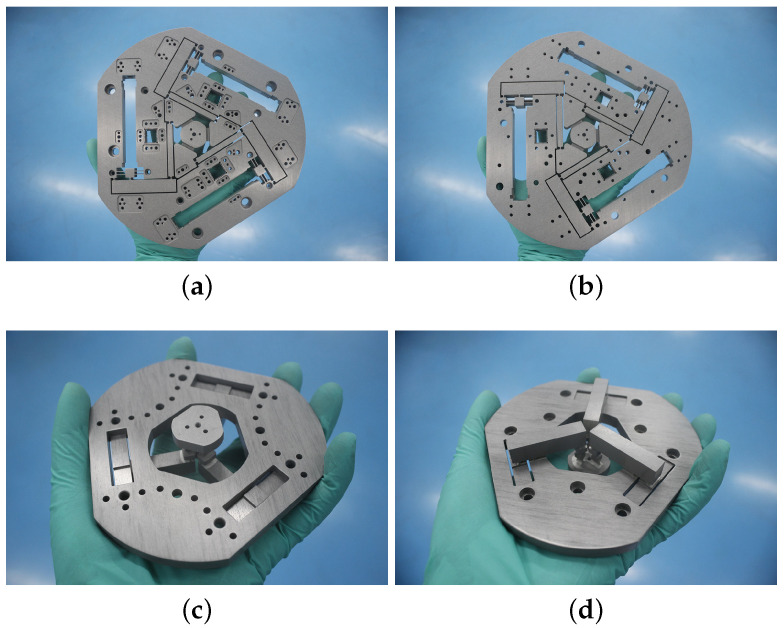
Fabricated prototypes of the two-in-one 6-RRRR CPM. (**a**) In-plane 3-RRRR CPM; (**b**) bottom-to-up view; (**c**) out-of-plane 3-RRRR CPM; (**d**) bottom-to-up view.

WEDM and CNC technology were employed to fabricate the in-plane 3-RRRR CPM and out-of-plane 3-RRRR CPM. The two CPMs were fabricated from the aluminum alloy AA 7075-T651. The machining method is mature and has been widely applied. The manufacturing procedure is simple and affordable. Dimension accuracy and uniform rigidity are guaranteed. The two fabricated prototypes are shown in Figure 15.

Figure 15a shows a top-to-bottom view of the upper substructure, which is the in-plane 3-RRRR CPM. Figure 15b shows a bottom-to-top view of the substructure shown in Figure 15a. Figure 15c shows a top-to-bottom view of the out-of-plane 3-RRRR CPM, and Figure 15d shows a bottom-to-top view of the substructure shown in Figure 15c. The in-plane and out-of-plane CPMs were separately fabricated. The two CPMs were connected by using a metal plate, several screws, and dowels.

#### 5.1.2. Instrumental Setup

Six PEAs were used to drive the six branched chains of the 6-RRRR NPS. Three packaged PEAs (P-841.3B, Physik Instrumente (PI) GmbH & Co. KG, Karlsruhe, Germany) possessed a maximum elongation of 45.0 μm. The static stiffness of the PEA was 19.0 N/μm ± 20%, and the dynamic stiffness was 19.0 N/μm ± 30%. For each packaged PEA, an SGS sensor was embedded to measure the elongation, with a sampling frequency of 2.00 kHz and a resolution of 0.45 nm. These PEAs drive the formation of the three in-plane branched chains. Three naked PEAs (NAC2015-H28, Piezomechanik GmbH, Munich, Germany) possess an open-loop maximum elongation of 42.9 μm. The stiffness is 98.0 N/μm. Without the embedded SGS sensors, the three naked PEAs have smaller sizes. The naked PEAs were employed to drive the three out-of-plane branched chains.

**Figure 16:**
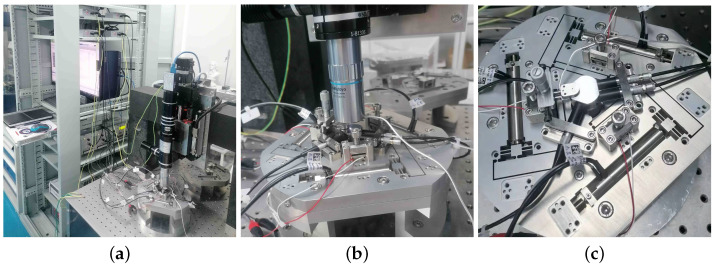
Instrumental setup to test the prototype of the 6-RRRR NPS. (**a**) Complete setup; (**b**) NPS setup; (**c**) sensors and PEAs.

As shown in Figure 16c, three cylindrical capacitive sensors (D-E 20.200, from PI GmbH) and one flaky capacitive sensor (D-E 30.200, from PI GmbH) were employed to measure the displacements of the end-effector. A controller board (MicroLabBox, dSPACE GmbH, Paderborn, Germany) produced six driving signals and processed the measured data. The prototype, PEAs, and sensors were fixed onto a vibration isolation desk.

### 5.2. Verification of the Kinematics Performance

The Jacobian matrix between the six actuators and output displacements is the core of the kinematics. The accuracy of the Jacobian matrix is assessed using the workspace and trajectory tracking precision as the two indices.

#### 5.2.1. Workspace

**Figure 17:**
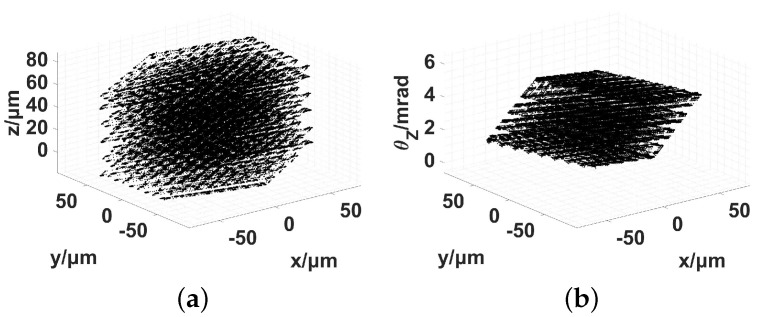
Workspace of the 6-RRRR NPS using a 3D view. (**a**) x−y−z; (**b**) x−y−θz.

The three translational DOFs and one in-plane rotational DOF were the four prior-focused aspects during the workspace test. The maximum elongation of the three in-plane packaged PEAs was 45.0 μm. The maximum driving voltage of the three out-of-plane naked PEAs is 150 V. The measured workspace is illustrated in Figure 17.

**Figure 18:**
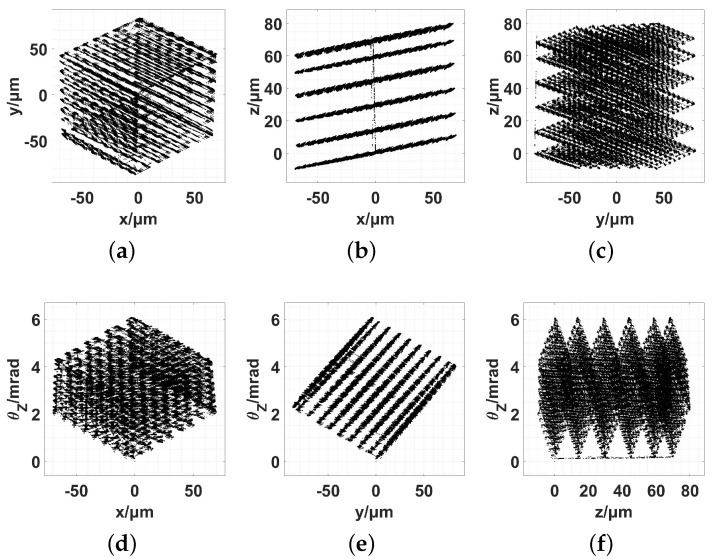
Workspace of the 6-RRRR NPS using the 2D view. (**a**) x−y; (**b**) x−z; (**c**) y−z; (**d**) x−θz; (**e**) y−θz; (**f**) z−θz.

The workspace was clearly defined by employing two-dimensional (2D) views, as shown in Figure 18.

The boundary results of the workspace obtained using the different methods are listed in Table 9.

#### 5.2.2. In-Plane Trajectory Tracking

**Figure 19:**
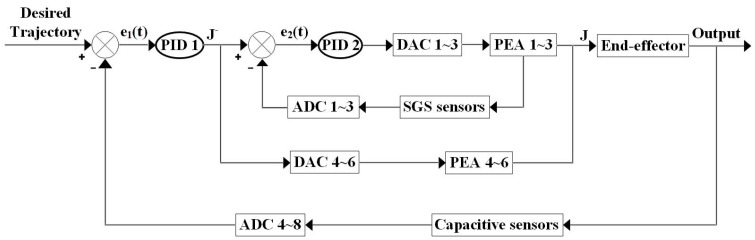
Controller architecture for in-plane trajectory tracking.

The in-plane trajectory was defined in three parts: a left-to-right line of 25.0 μm, a circle with a diameter of 50.0 μm, and a right-to-left line of 25.0 μm. Before the trajectory, two preparation steps were required to be completed. The first step is the sensor signal processing. The second step was rotation at the original point with half of the entire rotational stroke. After the trajectory, the rotational angle is reduced to zero. Two proportional-integral-derivative (PID) controllers were employed in the three packaged PEAs and the end-effector, as shown in Figure 19.

Four capacitive sensors were embedded into the NPS to measure the four DOFs of the end effector. To describe the coupling characteristics quantitatively, the in-plane rotational DOF and out-of-plane DOFs of the end-effector were not included in the first PID controller. For the two in-plane translational DOFs of the end-effector, the servo parameters of the first PID controller were set as $k_{p}=0.50$ and $k_{i}=1.0$.

**Figure 20:**
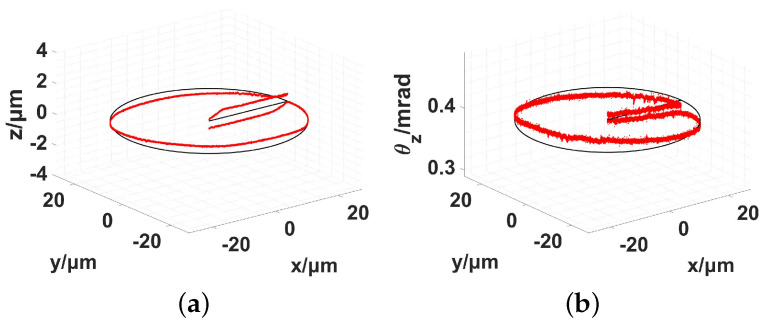
Measured trajectory during in-plane circle tracking. (**a**) x−y−z; (**b**) x−y−θz.

**Figure 21:**
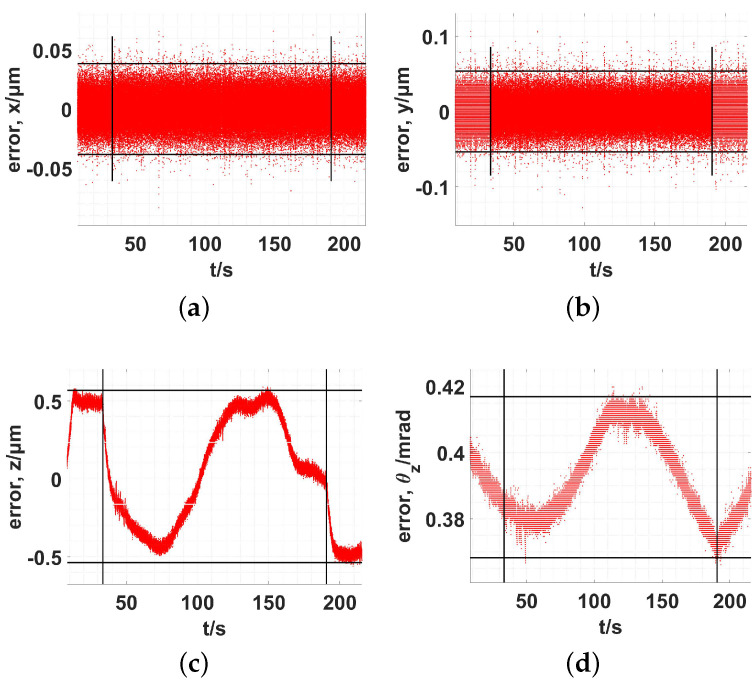
Tracking error of the in-plane trajectory and the coupling error. (**a**) x; (**b**) y; (**c**) z; (**d**) θz.

For the three in-plane PEAs, the servo parameters of the second PID controller were set to $k_{p}=0.80$ and $k_{i}=0.10$. The out-of-plane DOFs of the end effector were not included in the first PID controller. Therefore, the corresponding three out-of-plane PEAs were not included in the second PID controller. The tracking results are presented in Figure 20 and Figure 21.

The tracking speed of the end-effector was set to 1.00 μm/s, and the results using 3σ (σ, standard error) are shown in Table 10.

Without active control during the out-of-plane DOFs, the in-plane movements would result in large output coupling along the out-of-plane DOFs. The coupling translational displacement reached a maximum of 568 nm and minimum of −536 nm. The coupling rotational angle varied between 417 μrad and 368 μrad. The tracking experiment was based on a constant-parameter inverse kinematic Jacobian matrix. The elements of the Jacobian matrix are average values. Every point of the circular trajectory has a different Jacobian matrix, irrespective of whether it is forward or inverse. The kinematic relationship was based on two simplified conditions.

### 5.3. Comparison of the Design and Performance of NPSs

The three-dimensional size of the entire NPS was selected to describe the design of the mechanism. The workspace, natural frequency, and positioning accuracy were selected to represent the mechanical performance. Table 11 compares this NPS with similar previously published NPSs [35,36,37,38,39].

In Table 11, “WLH” represents the width, length, and height of the NPS; “123” represents the first-, second-, and third-order natural frequency; and “accuracy” selects the circle tracking results along the x-axis using the 3σ principle.

### 5.4. Discussion of Results

(1) The first focus is on mechanism design. The six parallel-kinematic branched chains of the 6-RRRR CPM were within the specified three-dimensional constraint of ϕ200 × 44 mm${}^{3}$. Equipped with six actuators and four sensors, the external dimensions of the 6-RRRR NPS were ϕ200 × 56 mm${}^{3}$. As indicated in Table 11, the proposed 6-RRRR NPS has a relatively concise and compact mechanical structure.

(2) The second focus is on the mechanical performance. The workspace of the 6-RRRR NPS was verified as 140 μm (x) × 170 μm (y) × 90.4 μm (z) × 6090 μrad (θz). In particular, the proposed NPS is advantageous for the in-plane rotational stroke (θz). The static and dynamic results of the 6-RRRR CPM verify the analysis method using CMM and PRBM. Both the output stiffness and natural frequency are advantageous. Along a circle of ϕ50.0 μm, the trajectory tracking accuracy was 38.4 nm (x, 3σ) and 53.6 nm (y, 3σ).

(3) Unfortunately, some of the coupling ratios of the 6-RRRR CPM were high, and this characteristic cannot be neglected. For the 6-RRRR NPS, the coupling ratios decrease to a low level. An in-plane circular trajectory with a diameter of 50.0 μm was tested. The coupling translational displacement ranged from −536 nm to 568 nm and the coupling rotational angle ranged from 368 μrad to 417 μrad. Further structural optimization and experimental verification would have to be conducted in future work.

(4) Owing to the new concise structure and verified high performance, the 6-RRRR NPS provides a new balanced solution to overcome the structural complexity of spatial multi-DOF NPSs, and to ensure fabrication feasibility and superior mechanical performance.

## 6. Conclusions

A 6-RRRR CPM with a novel two-in-one configuration is proposed in this paper. The configuration addresses the first challenge of the mechanism design to balance fabrication feasibility and mechanical performance. The prototypes were easily fabricated using traditional WEDM and CNC at low cost. Simultaneously, the mechanical performance was verified using FEM and experiments.

The second challenge, which related to the analysis of the mechanical performance, involved balancing the calculation accuracy and computational time. This challenge was addressed using the CMM and PRBM to develop a method for high-efficiency performance analysis proposed in this paper. For static performance, a model with 85 nodes and 84 frames using the CMM was presented. The simulation results for the stiffness and coupling ratio verified the accuracy of the calculation. For the dynamic performance, a simplified equivalent model was built using the simulation results of the natural frequency. A Jacobian matrix was derived for the kinematic performance. The mapping relationship was verified using the experimental results of workspace and trajectory tracking.

The verified performance demonstrates the advantages of the presented 6-RRRR CPM, fulfilling the demands of both the challenges. Relative to spatial multi-DOF NPSs from available commercial goods or published papers, the advantages of the 6-RRRR NPS are its low manufacturing cost and superior mechanical performance.

Future research intends to focus on the experimental verification of the static and dynamic performance, powerful control algorithms to reinforce the mechanical performance, a more precise mathematical model with less simplified conditions, and the two other rotational DOFs of the end-effector with two more capacitive sensors.

## Figures and Tables

**Table 1:** Input stiffness along six branched chains of the 6-RRRR CPM.

$k_{in}^{j2}$ (N/μm)	$j_{2}$					
	1	16	31	46	59	72
CMM	5.22	3.89	3.78	6.91	6.89	6.93
FEM	5.35	4.29	4.08	6.34	6.32	6.30
Error	2.4%	9.4%	7.4%	9.0%	9.0%	9.9%

**Table 2:** Output stiffness along four DOFs of the 6-RRRR CPM.

$k_{out}^{j3}$	$j_{3}$							
	z (N/μm)		x (N/μm)		y (N/μm)		θz *	
	min	max	min	max	min	max	min	max
CMM	0.81	5.68	0.42	2.03	0.46	1.94	0.36	2.13
FEM	0.86	6.25	0.47	2.24	0.52	2.24	0.32	2.58
Error	5.3%	9.1%	11%	9.2%	12%	13%	15%	17%

Note: * = N · mm/μrad.

**Table 3:** Output stiffness of the 6-RRRR NPS using the FEM.

$k_{out}^{j3}$	$j_{3}$			
	z (N/μm)	x (N/μm)	y (N/μm)	θz (N·mm/μrad)
FEM	5.28	1.27	1.32	1.28

**Table 4:** Input coupling ratio of the 6-RRRR CPM using the FEM.

$c_{\mathrm{in}}^{j_{21}j_{22}}$		$j_{22}$					
		1st	2nd	3rd	4th	5th	6th
$j_{21}$	1st	/	17%	17%	3.2%	4.5%	4.5%
	2nd	13%	/	21%	6.0%	2.7%	5.9%
	3rd	14%	20%	/	6.2%	5.8%	2.6%
	4th	5.0%	9.5%	10%	/	63%	63%
	5th	6.5%	5.8%	9.8%	63%	/	62%
	6th	6.5%	9.3%	5.5%	63%	62%	/

**Table 5:** Input coupling ratio of the 6-RRRR NPS using the FEM.

$c_{\mathrm{in}}^{j_{21}j_{22}}$		$j_{22}$					
		1st	2nd	3rd	4th	5th	6th
$j_{21}$	1st	/	3.2%	3.1%	1.1%	0.83%	0.83%
	2nd	3.1%	/	4.5%	1.3%	0.63%	1.0%
	3rd	3.3%	4.2%	/	1.3%	1.0%	0.64%
	4th	3.3%	5.9%	5.8%	/	4.9%	4.9%
	5th	3.7%	4.0%	4.8%	4.9%	/	4.4%
	6th	3.9%	4.6%	3.8%	4.9%	4.4%	/

**Table 6:** Output coupling ratio of the 6-RRRR CPM using the FEM.

$c_{\mathrm{out}}^{j_{31}j_{32}}$		$j_{32}$					
		z	x	y	θz	θx	θy
$j_{31}$	z	/	0.89%	0.60‰	0.006 *	/	/
	x	0.21%	/	1.2%	0.003 *	0.017 *	1.382 *
	y	4.1%	1.3%	/	0.004 *	1.204 *	0.017 *
	θz	0.15 **	1.51 **	8.72 **	/	3.3%	1.2%

Note: * = mrad/(50.0 μm); ** = μm/(2.95 mrad).

**Table 7:** Output coupling ratio of the 6-RRRR NPS using the FEM.

$c_{\mathrm{out}}^{j_{31}j_{32}}$		$j_{32}$					
		z	x	y	θz	θx	θy
$j_{31}$	z	/	0.39‰	0.87‰	0.005 *	/	/
	x	0.43‰	/	4.3‰	0.006 *	0.013 *	1.567 *
	y	0.23‰	3.8‰	/	0.001 *	1.551 *	0.006 *
	θz	0.07 **	0.18 **	1.64 **	/	3.8‰	0.25‰

Note: * = mrad/(50.0 μm); ** = μm/(2.95 mrad).

**Table 8:** Natural frequency of the 6-RRRR CPM and NPS using the FEM.

Natural Frequency			1st	2nd	3rd	4th
6-RRRR	NPS	/	725.70	734.41	873.44	948.24
	CPM	max	947.92	976.46	1045.7	1085.2
	CPM	min	399.67	425.61	460.24	478.30
3-RRRR, i	CPM	min	426.96	446.85	467.46	546.65
3-RRRR, o	CPM	min	439.30	475.27	664.77	1614.9

Note: 3-RRRR, i = in-plane 3-RRRR CPM; 3-RRRR, o = out-of-plane 3-RRRR CPM.

**Table 9:** Comparison of the workspace in terms of the kinematics performance.

DOF	z (μm)		x (μm)		y (μm)		θz (mrad)	
	min	max	min	max	min	max	min	max
PRBM	/	91.0	−67.2	69.6	−81.5	79.0	0.00	5.63
Test	−10.1	80.3	−69.9	70.0	−86.8	83.6	0.00	6.09

**Table 10:** Tracking the two planed DOFs and coupling of two other DOFs.

Error	x (nm)	y (nm)	z Coupling (nm)		θz Coupling (μrad)	
	3σ	3σ	min	max	min	max
Value	38.4	53.6	−536	568	368	417

**Table 11:** Comparison of selected indexes of the mechanism design and mechanical performance.

NPS	Dimension	Workspace		Frequency	Accuracy
	WLH/mm${}^{3}$	xyz/μm${}^{3}$	θz/μrad	123/Hz${}^{3}$	3σ/nm
Ref. [35]	250 × 250 × 80	40 × 40 × 80	400	200	33
Ref. [36]	ϕ150 × 143	8 × 10 × 13	200	323 × 323 × 526	99
Ref. [37]	380 × 380 × 115	100 × 100 × 300	500	54 × 55 × 75	/
Ref. [38]	ϕ264 × 148	80 × 80 × 60	300	189 × 189 × 231	30
Ref. [39]	241 × 241 × 67	111 × 111 × 260	2700	41 × 46 × 48	/
6-RRRR	ϕ200 × 56	140 × 170 × 90	6090	726 × 734 × 873	38

Note: the coupling translational displacement of the 6-RRRR NPS ranged from −536 nm to 568 nm; the coupling rotational angle ranged from 368 μrad to 417 μrad.

## Data Availability

Research data are available from the authors.

## Abbreviations

The following abbreviations are used in this manuscript:

PEA	Piezoelectric actuator
CPM	Compliant parallel mechanism
NPS	Nanopositioning stage
RRRR	revolute-revolute-revolute-revolute
3-RRR	3-revolute-revolute-revolute
6-RRRR	3-revolute-revolute-revolute-revolute
CMM	Compliance-based matrix method
PRBM	Pseudo-rigid body model
CSM	Compliant serial mechanism
CHM	Compliant serial-parallel hybrid mechanism
FEM	Finite element method
SaNFH	Single-axis notch flexure hinge
WEDM	Wire electrical discharge machining
CNC	Computer numerical control

## References

[1] Ryu J.W., Gweon D.G., Moont K.S. (1997). Optimal design of a flexure hinge based XY*θ* wafer stage. Precis. Eng..

[2] Chang S.H., Tseng C.K., Chien H.C. (1999). An ultra-precision XY*θ*_Z_ piezo-micropositioner part I: Design and analysis. IEEE Trans. Ultrason. Ferroelectr. Freq. Control.

[3] Yong Y.K., Lu T.F., Handley D.C. (2004). Loop Closure Theory in Deriving Linear and Simple Kinematic Model for a 3-DOF Parallel Micromanipulator. Proc. SPIE.

[4] Yong Y.K., Lu T.F. (2009). Kinetostatic modeling of 3-RRR compliant micro-motion stages with flexure hinges. Mech. Mach. Theory.

[5] Tian Y., Shirinzadeh B., Zhang D. (2010). Design and dynamics of a 3-DOF flexure-based parallel mechanism for micro/nano manipulation. Microelectron. Eng..

[6] Qin Y., Shirinzadeh B., Zhang D., Tian Y. (2013). Design and Kinematics Modeling of a Novel 3-DOF Monolithic Manipulator Featuring Improved Scott-Russell Mechanisms. J. Mech. Des..

[7] Bhagat U., Shirinzadeh B., Clark L., Chea P., Qin Y., Tian Y., Zhang D. (2014). Design and analysis of a novel flexure-based 3-DOF mechanism. Mech. Mach. Theory.

[8] Guo Z., Tian Y., Liu C., Wang F., Liu X., Shirinzadeh B., Zhang D. (2015). Design and control methodology of a 3-DOF flexure-based mechanism for micro/nano-positioning. Robot. Comput.-Integr. Manuf..

[9] Wang R., Zhang X. (2016). A planar 3-DOF nanopositioning platform with large magnification. Precis. Eng..

[10] Wang R., Zhang X. (2017). Optimal design of a planar parallel 3-DOF nanopositioner with multi-objective. Mech. Mach. Theory.

[11] Ding B., Li Y., Xiao X., Tang Y., Li B. (2017). Design and analysis of a 3-DOF planar micromanipulation stage with large rotational displacement for micromanipulation system. Mech. Sci..

[12] Wang R., Zhang X. (2018). Parameters Optimization and Experiment of A Planar Parallel 3-DOF Nanopositioning System. IEEE Trans. Ind. Electron..

[13] Gu Y., Chen X., Lu F., Lin J., Yi A., Feng J., Sun Y. (2019). Development of a Novel Three Degrees-of-Freedom Rotary Vibration-Assisted Micropolishing System Based on Piezoelectric Actuation. Micromachines.

[14] Yu H., Zhang C., Yang B., Chen S.L., Fang Z., Li R., Yang G. (2019). The design and kinetostatic modeling of 3PPR planar compliant parallel mechanism based on compliance matrix method. Rev. Sci. Instruments.

[15] Kim H.Y., Ahn D.H., Gweon D.G. (2012). Development of a novel 3-degrees of freedom flexure based positioning system. Rev. Sci. Instruments.

[16] Park J., Lee H., Kim H., Kim H., Gweon D. (2016). Note: Development of a compact aperture-type XY*θ*_z_ positioning stage. Rev. Sci. Instruments.

[17] Ando T., Kodera N., Takai E., Maruyama D., Saito K., Toda A. (2001). A high-speed atomic force microscope for studying biological macromolecules. Proc. Natl. Acad. Sci. USA.

[18] Kenton B.J., Leang K.K. Design, Characterization, and Control of a Monolithic Three-Axis High-Bandwidth Nanopositioning Stage. Proceedings of the American Control Conference (ACC).

[19] Yue Y., Gao F., Zhao X., Ge Q.J. (2010). Relationship among input-force, payload, stiffness and displacement of a 3-DOF perpendicular parallel micro-manipulator. Mech. Mach. Theory.

[20] Li Y., Xu Q. (2011). A Totally Decoupled Piezo-Driven XYZ Flexure Parallel Micropositioning Stage for Micro/Nanomanipulation. IEEE Trans. Autom. Sci. Eng..

[21] Kenton B.J., Leang K.K. (2012). Design and Control of a Three-Axis Serial-Kinematic High-Bandwidth Nanopositioner. IEEE/ASME Trans. Mechatronics.

[22] Hao G., Kong X. (2012). Design and Modeling of a Large-Range Modular XYZ Compliant Parallel Manipulator Using Identical Spatial Modules. J. Mech. Robot..

[23] Awtar S., Parmar G. (2013). Design of a Large Range XY Nanopositioning System. J. Mech. Robot..

[24] Yong Y.K., Bhikkaji B., Moheimani S.O.R. (2013). Design, Modeling, and FPAA-Based Control of a High-Speed Atomic Force Microscope Nanopositioner. IEEE/ASME Trans. Mechatronics.

[25] Yong Y.K., Moheimani S.O.R. (2015). Collocated Z-Axis Control of a High-Speed Nanopositioner for Video-Rate Atomic Force Microscopy. IEEE Trans. Nanotechnol..

[26] Zhou C., Gong Z., Chen B.K., Cao Z., Yu J., Ru C., Tan M., Xie S., Sun Y. (2016). A Closed-Loop Controlled Nanomanipulation System for Probing Nanostructures Inside Scanning Electron Microscopes. IEEE/ASME Trans. Mechatronics.

[27] Tang C., Zhang M., Cao G. (2017). Design and testing of a novel flexure-based 3-degree-of-freedom elliptical micro/nano-positioning motion stage. Adv. Mech. Eng..

[28] Watanabe S., Ando T. (2017). High-speed XYZ-nanopositioner for scanning ion conductance microscopy. Appl. Phys. Lett..

[29] Zhang X., Xu Q. (2018). Design and Testing of a New 3-DOF Spatial Flexure Parallel Micropositioning Stage. Int. J. Precis. Eng. Manuf..

[30] Ling M., Cao J., Li Q., Zhuang J. (2018). Design, pseudo-static model and PVDF-based motion sensing of a piezo-actuated XYZ flexure manipulator. IEEE/ASME Trans. Mechatronics.

[31] Ghafarian M., Shirinzadeh B., Al-Jodah A., Das T.K., Wei W., Tian Y., Zhang D. (2020). An XYZ micromanipulator for precise positioning applications. J. Micro-Bio Robot..

[32] Tian Y., Lu K., Wang F., Zhou C., Ma Y., Jing X., Yang C., Zhang D. (2020). A Spatial Deployable Three-DOF Compliant Nano-Positioner With a Three-Stage Motion Amplification Mechanism. IEEE/ASME Trans. Mechatronics.

[33] Wadikhaye S., Yong Y., Moheimani S. (2012). Design of a compact serial-kinematic scanner for high-speed atomic force microscopy: An analytical approach. Micro Nano Lett..

[34] Wadikhaye S.P., Yong Y.K., Moheimani S.O.R. (2014). A serial-kinematic nanopositioner for high-speed atomic force microscopy. Rev. Sci. Instruments.

[35] Varadarajan K.M., Culpepper M.L. (2007). A dual-purpose positioner-fixture for precision six-axis positioning and precision fixturing: Part II. Characterization and calibration. Precis. Eng..

[36] Cai K., Tian Y., Liu X., Fatikow S., Wanga F., Cui L., Zhang D., Shirinzadeh B. (2018). Modeling and controller design of a 6-DOF precision positioning system. Mech. Syst. Signal Process..

[37] Shin H.P., Moon J.H. (2018). Static and Dynamic Analyses of a 6-DOF Ultra-Precision Parallel Mechanism. Int. J. Precis. Eng. Manuf..

[38] Zhang D., Li P., Zhang J., Chen H., Guo K., Ni M. (2019). Design and Assessment of a 6-DOF Micro/Nanopositioning System. IEEE/ASME Trans. Mechatronics.

[39] Lin C., Zheng S., Jiang M. (2020). Dynamic Analysis and Experiment of 6-DOF Compliant Platform Based on Bridge-Type Amplifier. Micromachines.

[40] Chang Q., Gao X., Liu Y., Deng J., Zhang S., Chen W. (2022). Development of a cross-scale 6-DOF piezoelectric stage and its application in assisted puncture. Mech. Syst. Signal Process..

[41] Liao S., Ding B., Li Y. (2022). Design, Assembly, and Simulation of Flexure-Based Modular Micro-Positioning Stages. Machines.

